# Long Noncoding RNA TUG1/miR-29c Axis Affects Cell Proliferation, Invasion, and Migration in Human Pancreatic Cancer

**DOI:** 10.1155/2018/6857042

**Published:** 2018-11-22

**Authors:** Yebin Lu, Ling Tang, Zhipeng Zhang, Shengyu Li, Shuai Liang, Liandong Ji, Bo Yang, Yu Liu, Wei Wei

**Affiliations:** ^1^Department of General Surgery, Xiangya Hospital, Central South University, Changsha 410008, China; ^2^Department of Pharmacy, Xiangya Hospital, Central South University, Changsha 410008, China; ^3^Department of Vascular Surgery, Tianjin First Center Hospital, 300192, China; ^4^Department of Pathology, Hunan Provincial People's Hospital, Changsha 410005, China

## Abstract

Given the low resection rate and chemoresistance of patients with pancreatic cancer (PC), their survival rates are typically poor. Long noncoding RNAs (lncRNAs) have recently been shown to play an important role in tumourigenesis and human cancer progression, including in PC. In this study, we aimed to investigate the role of taurine-upregulated gene 1 (TUG1) in PC. A quantitative polymerase chain reaction was used to analyse TUG1 expression in PC tissues and peritumoural normal tissues. TUG1 was overexpressed in PC tissues compared with that in peritumoural normal tissues, and the high expression of TUG1 was associated with the poor prognosis of patients with PC. Furthermore, TUG1 knockdown significantly inhibited the proliferation and invasion of PC cells both in vitro and in vivo, while overexpression TUG1 promoted tumour cell proliferation, migration, and invasion. TUG1 directly targeted miR-29c, a tumour suppressor in several cancers. TUG1 knockdown significantly increased the expression of miR-29c and subsequently induced the downregulation of integrin subunit beta 1 (ITGB1), matrix metalloproteinase-2 (MMP2), and matrix metalloproteinase-9 (MMP9). The downregulation of miR-29c abolished the TUG1 knockdown-mediated inhibition of tumour growth in vitro and in vivo, whereas the upregulation of miR-29c enhanced the effects of TUG1 knockdown on PC cells. In conclusion, we demonstrate for the first time the oncogenic role of TUG1 in PC. The downregulation of TUG1 significantly inhibited the growth and migratory ability of PC cells in vitro and in vivo by targeting miR-29c. Our study provides a novel potential diagnostic biomarker and therapeutic target for PC.

## 1. Background

In humans, protein-coding genes account for approximately 2% of the genome, whereas the vast majority are noncoding RNAs, including microRNAs and long noncoding RNAs (lncRNAs) [1]. In recent years, research on lncRNAs has evoked considerable interest. lncRNAs function as regulatory molecules in a wide range of biological processes [2] and play an important role in tumourigenesis and human cancer progression. The dysregulation of lncRNAs is well documented in the context of several types of cancers, including breast cancer, hepatocellular cancer, nasopharyngeal carcinoma, and pancreatic cancer (PC) [3].

Patients with PC tend to have poor prognosis because of chemoresistance and typically low resection rates. Hence, early diagnosis and treatment are critical for the management of PC. Therefore, new biomarkers for diagnosis and prognostic assessment are urgently needed. Recent research has unravelled the role of lncRNAs in carcinogenesis via the regulation of cell proliferation, migration, invasion, metastasis, and chemoresistance [4]. Several studies have revealed that some lncRNAs involved in biological functions are dysregulated in PC. lncRNA urothelial cancer-associated 1 (UCA1) was shown to play a pivotal role in bladder cancer progression and embryonic development. The downregulation of UCA1 was shown to inhibit cell proliferation, induce apoptosis, and cause cell cycle arrest in PC cells [5]. lncRNA MALAT1 was found to be highly expressed in pancreatic ductal adenocarcinoma tissues, and its elevated expression was associated with poor prognosis. lncRNA MALAT1 is believed to regulate tumourigenesis via HuR-TIA-1-mediated autophagic activation [6]. The lncRNA LINC00673, which is a potential tumour suppressor, is associated with PC risk and plays an important role in maintaining cell homeostasis in PC [7].

More recently, lncRNA taurine-upregulated gene 1 (TUG1) was identified as an oncogenic lncRNA. The aberrant upregulation of TUG1 has been documented in different types of cancer, including B-cell malignancies, bladder cancer, hepatocellular carcinoma, and osteosarcoma [8]. TUG1 expression was also shown to be significantly upregulated in gallbladder carcinoma tissues. TUG1 knockdown significantly inhibited gallbladder cancer cell proliferation and metastasis via the inhibition of epithelial-mesenchymal transition (EMT) [9]. Furthermore, TUG1 knockdown was shown to significantly inhibit the proliferation, migration, and invasion of colorectal cancer cells in vitro [10]. Conversely, TUG1 is generally downregulated in non-small-cell lung carcinoma (NSCLC) tissues. A lower expression of TUG1 was associated with a higher TNM stage and tumour size, as well as poorer overall survival for patients with NSCLC. TUG1 knockdown was shown to significantly promote the proliferation of NSCLC cancer cells in vitro and in vivo [11]. These findings indicate a tissue-specific function of TUG1 in the context of tumourigenesis. Interestingly, TUG1 expression in pancreatic tissues was shown to be higher than that in other organ tissues, and the expression levels were dynamically regulated by glucose in Nit-1 cells. The knockdown of TUG1 expression resulted in an increased apoptosis ratio and decreased insulin secretion in *β*-cells both in vitro and in vivo [12]. These findings suggest that TUG1 has an important role in the pathological and physiological processes of pancreatic cells. However, the role of TUG1 in the genesis of PC, as well as the associated underlying mechanisms, has not been elucidated.

In our previous study, we found that miR-29c inhibits the growth, invasion, and migration of PC cells by targeting integrin subunit beta 1 (ITGB1) [13]. In the present study, we aimed to investigate the role of TUG1 in PC. We found that TUG1 was overexpressed in PC tissues and that TUG1 knockdown significantly inhibited cell proliferation and invasion of PC in vitro and in vivo. Furthermore, we investigated the underlying mechanisms by which TUG1 knockdown inhibits PC cell growth.

## 2. Materials and Methods

### 2.1. Study Population

A total of 72 surgical specimens of pancreatic cancer tissues and 20 samples of peritumoural normal tissues were collected from the Department of General Surgery, Xiangya Hospital, Central South University. All tissues were formalin-fixed and paraffin-embedded and were stored at 4°C before usage. The age of the pancreatic cancer patients (45 males and 27 females) ranged from 28 years to 76 years. The clinical characteristics of the patients were retrieved from the medical records. None of the patients had received any therapy prior to sample collection. The present study was approved by the Ethics Committee of Xiangya Hospital of Central South University. Written informed consent was obtained from all participants involved in this study.

### 2.2. Cell Culture and Treatment

Human pancreas ductal epithelioid (HPDE) cells and four human pancreatic cancer cell lines (SW1990, AsPC-1, BxPC-3, and PANC-1) were purchased from the American Type Culture Collection. Cells were grown in RPMI-1640 medium (Invitrogen, CA, USA) supplemented with 10% fetal bovine serum (Gibco, CA, USA) and cultured in a 37°C humidified atmosphere of 5% CO_2_. The knockdown and overexpression of TUG1 in BxPC-3 and PANC-1 cells were achieved by transfection with lentivirus vector containing TUG1 shRNA (forward, 5′-GATCCGCTTGGCTTCTATTCTGAATCCTTTCAAGAGAAGGATTCAGAATAGAAAGCCAAGCCAAGCTTTTTTG-3′; reverse, 5′-GCGAACCGAAGATAAGACTTAGGAAAGTTCTCTTCCTAAGTCTTATCTTCGGTTCGAAAAAAC-3′; GenePharma, Shanghai, China), The overexpression of TUG1 in SW1990 cells was achieved by transfection with the TUG1-pcDNA3.1 plasmid which constructed by Invitrogen (Invitrogen, CA, USA). Cells were transfected by Lipofectamine 2000 (Invitrogen, CA, USA). The overexpression and knockdown of miR-29c were performed using miR-29c mimic and miR-29c inhibitor (GeneCopoeia, Guangzhou, China), respectively. Cells transfected with empty vector or scramble control were used as negative control. Cells were plated in 6-well clusters or 96-well plates and transfected for 24 or 48 h. Transfected cells were used for further assays or protein extraction.

### 2.3. RNA Extraction and qRT-PCR Analysis

Total RNA was extracted from cells by using TRIzol reagent (Invitrogen, CA, USA). miR-29c expression in cells was detected using a Hairpin-it TM miRNAs qPCR kit (GenePharma, Shanghai, China) according to the manufacturers' instructions. The expression of RNU6B was used as an endogenous control. The expression of TUG1 was measured by SYBR Green qPCR assay (Takara, Dalian, China) according to the manufacturers' instructions. Primers were designed by Shanghai Sangon Biotech Co. Ltd. (TUG1 F: 5′-TAGCAGTTCCCCAATCCTTG-3′; R: 5′-CACAAATTCCCATCATTCCC-3′). The expression of *β*-actin was used as an endogenous control. qRT-PCR was performed under the following conditions: 95.0°C for 3 min, 39 cycles of 95.0°C for 10 s and 60°C for 30 s. Data were processed using the 2^−ΔΔCT^ method.

### 2.4. Cell Counting Kit-8 Cell Proliferation Assay

The cell proliferation rates were measured using cell counting kit-8 (CCK-8) (Beyotime, Hangzhou, China). Approximately 0.5 × 10^4^ cells were seeded in each 96-well plate for 24 h, transfected with the indicated plasmids, and further incubated for 24, 48, and 72 h. A total of 10 *μ*L CCK-8 reagents were added to each well at 1 h before the endpoint of incubation. The optical density (OD) at 490 nm in each well was determined by a microplate reader.

### 2.5. Cell Migration and Invasion Assay

Cell migration was assessed by wound healing assays. In brief, cells were seeded in six-well plates and cultured to 100% confluence. By using a sterile pipette tip, wounds were generated, and the cells were cultured for 48 h. Thereafter, the wound closure was assessed by Scion Image Software (Scion Corporation, Frederick, MD). For cell invasion assays, the matrigel invasion chambers (BD Biosciences) were used to assess cell invasion ability. Briefly, 1 × 10^5^ cells were seeded in the upper chamber with media containing 0.1% fetal bovine serum, whereas the lower chamber was filled with media containing 10% fetal bovine serum. After incubation for 48 h, the noninvading cells were removed with cotton swabs, and the cells that invaded through the membrane were stained with 0.1% crystal violet and imaged. Subsequently, the staining was dissolved by 5% acetic acid. OD at 570 nm in each well was determined by a microplate reader.

### 2.6. Luciferase Reporter Assay

The partial sequences of TUG1 3′-untranslated region (UTR), which contains the putative miR-29c-binding site, were amplified by PCR and constructed into psiCHECK-2 vector (Promega, Madison, WI) to generate wild-type TUG1 reporter (wt-TUG1). The GeneArt™ Site-Directed Mutagenesis System (Thermo Fisher Scientific) was used to produce mutant-type TUG1 reporter (mut-TUG1). All constructs were verified by DNA sequencing. The cells were plated in 96-well clusters and subsequently cotransfected with 100 ng constructs with miR-29c mimic or with miR-29c inhibitor. At 48 h after transfection, luciferase activity was detected using a dual-luciferase reporter assay system (Promega, Madison, WI) and normalised to Renilla activity.

### 2.7. Western Blot Analysis

Cultured or transfected cells were lysed in RIPA buffer with 1% PMSF. Western blot was performed on 10% SDS-PAGE by using Mini-PROTEAN® Tetra Cell Systems (Bio-Rad). Proteins were transferred onto polyvinylidene difluoride membranes (Immobilon, Millipore). Membranes were incubated overnight with ITGB1 rabbit monoclonal antibody (Cell Signaling), E-cadherin rabbit monoclonal antibody (Cell Signaling), N-cadherin rabbit monoclonal antibody (Abcam), Vimentin rabbit monoclonal antibody (Abcam), MMP9 rabbit monoclonal antibody (Cell Signaling), or MMP2 rabbit monoclonal antibody (Cell Signaling) at 1 : 1000 dilution or *β*-actin-specific antibody (Sigma-Aldrich) at 1 : 5000 dilution at 4°C. Signals were visualised using ECL substrates (Millipore, MA, USA).

### 2.8. Tumour Xenograft in Nude Mice

Animal experiments were approved by the Ethical Committee for Animal Research of Central South University. Nude mice (4–5 weeks old, male, *n* = 5 per group) were purchased from the Central Animal Facility of Central South University. To assess tumour growth, 200 mL of PANC-1 cells (2 × 10^6^) was subcutaneously injected into the left side of the back of each mouse. The tumour size was measured regularly and calculated using the formula 0.52 × L × W^2^ (L and W are the long and short diameters of the tumour, respectively). The animals were euthanised on day 30 after injection, and the tumours were removed and captured.

### 2.9. Statistical Analysis

Data are expressed as mean ± standard deviation (SD). SPSS 16.0 software (SPSS Inc., IL, USA) was used to perform statistical analysis. Student's *t*-test was used to analyse the differential expression of TUG1 and miR-29c between pancreatic cancer patients and adjacent controls. A chi-square test was used to analyse the association between the level of TUG1 and clinicopathological parameters. *P* values less than 0.05 were considered statistically significant.

## 3. Results

### 3.1. TUG1 Expression in PC Tissues Was Higher than That in Adjacent Control Tissues

**Figure 1:**
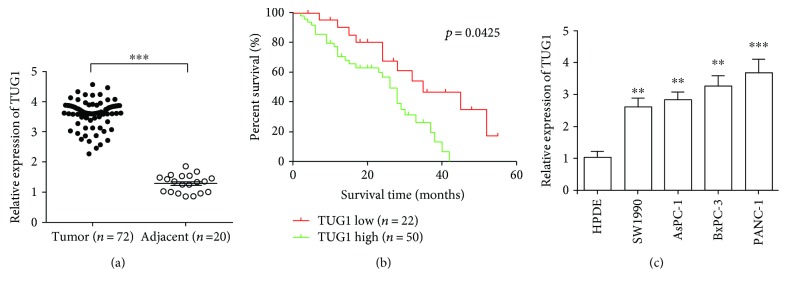
Long-noncoding RNA taurine-upregulated gene 1 (lncRNA TUG1) is increased in human pancreatic cancer (PC) tissues and cell lines. (a) qRT-PCR was used to analyse the expression of lncRNA taurine-upregulated gene 1 (TUG1). The expression of lncRNA TUG1 in human PC tissues was significantly higher than that in peritumoural normal tissues. (b) Kaplan-Meier analysis of overall survival stratified by low TUG1 expression (*n* = 22) and high TUG1 expression (*n* = 50). (c) qRT-PCR was used to analyse the expression of lncRNA TUG1 in cell lines. The expression of lncRNA TUG1 in human PC cell lines (SW1990, AsPC-1, BxPC-3, and PANC-1) was significantly higher than that in human pancreatic ductal epithelium cells. ^∗∗^ *P* < 0.01 and ^∗∗∗^ *P* < 0.001.

qRT-PCR performed to analyse TUG1 expression in 72 PC samples and 20 peritumoural normal tissues. TUG1 levels in PC tissues were significantly higher than those in adjacent tissues (Figure 1(a)). We further studied the association between TUG1 levels and clinical characteristics. All patients with PC were divided into two groups, namely, the high TUG1 level group (*n* = 50) and the low TUG1 level group (*n* = 22), on the basis of the mean expression level of TUG1 in adjacent tissues. As demonstrated in Table 1, the TUG1 level showed no correlation with age (*P* = 0.571), gender (*P* = 0.253), and tumour size (*P* = 0.159). However, it was significantly associated with lymph node metastasis (*P* = 0.019), pathological differentiation (*P* = 0.032), and clinical stage (*P* = 0.016). Kaplan-Meier survival analysis revealed that patients with high TUG1 expression had more poor overall survival (*P* < 0.05) than those with low TUG1 expression (Figure 1(b)). Moreover, we examined TUG1 expression levels in four PC cell lines (SW1990, AsPC-1, BxPC-3, and PANC-1); the relative expression levels of TUG1 in these PC cell lines were all significantly higher than those in the human pancreatic duct epithelial cells (Figure 1(c)). Our findings suggest that TUG1 levels may be used as a prognostic biomarker to assess the risk of malignancy progression in patients with PC.

### 3.2. Knockdown of TUG1 Inhibits PC Cell Proliferation, Invasion, and Migration

**Figure 2:**
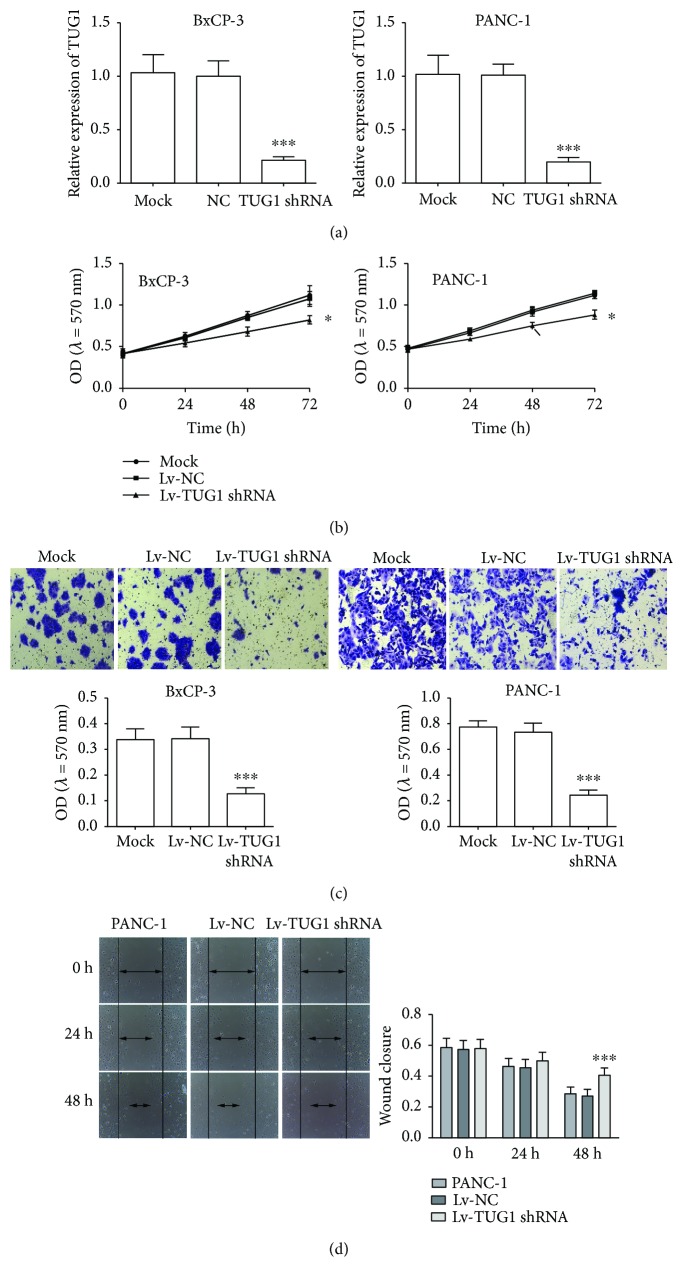
The knockdown of TUG1 represses the growth, invasion, and migration of BxPC-3 and PANC-1 cells. (a) qRT-PCR was used to determine the expression of lncRNA TUG1 in BxPC-3 (left) and PANC-1 (right) cells after Lv-TUG1 shRNA transfection. (b) CCK-8 was used to measure the proliferation of BxPC-3 (left) and PANC-1 (right) cells after Lv-TUG1 shRNA treatment. (c) Transwell assay was used to measure the invasive ability of BxPC-3 (left) and PANC-1 (right) cells after Lv-TUG1 shRNA treatment. (d) Wound healing assay was used to measure the migratory ability of PANC-1 cells after Lv-TUG1 shRNA treatment. Similar results were also obtained in BxPC-3 cells (data not shown). Data are expressed as mean ± SD. ^∗^ *P* < 0.05 and ^∗∗∗^ *P* < 0.001 vs. negative control.

To further investigate the biological role of TUG1 in PC progression, we infected BxPC-3 and PANC-1 cells with TUG1 shRNA vector and corresponding negative controls. The results of qRT-PCR revealed significant downregulation of TUG1 expression (Figure 2(a)). CCK-8 assays indicated that the proliferation of PC cells transfected with TUG1 shRNA was inhibited compared with NC and mock groups (Figure 2(b)). Transwell assay manifested that TUG1 knockdown inhibited the invasion of BxPC-3 and PANC-1 cells (Figure 2(c)). The scratch wound healing assay showed that the migration of BxPC-3 and PANC-1 cells treated with TUG1 shRNA was significantly suppressed with NC and mock groups, as indicated by a decrease in the closed wound area (Figure 2(d)). While enhancing the expression of TUG1 in SW1990 (Figure S1A), cell growth (Figure S1B), invasion (Figure S1C), and migration (Figure S1D) were promoted. These results suggested that knockdown of TUG1 significantly inhibited the growth, invasion, and migration of PCa cells.

### 3.3. TUG1 Interacts with miR-29c

**Figure 3:**
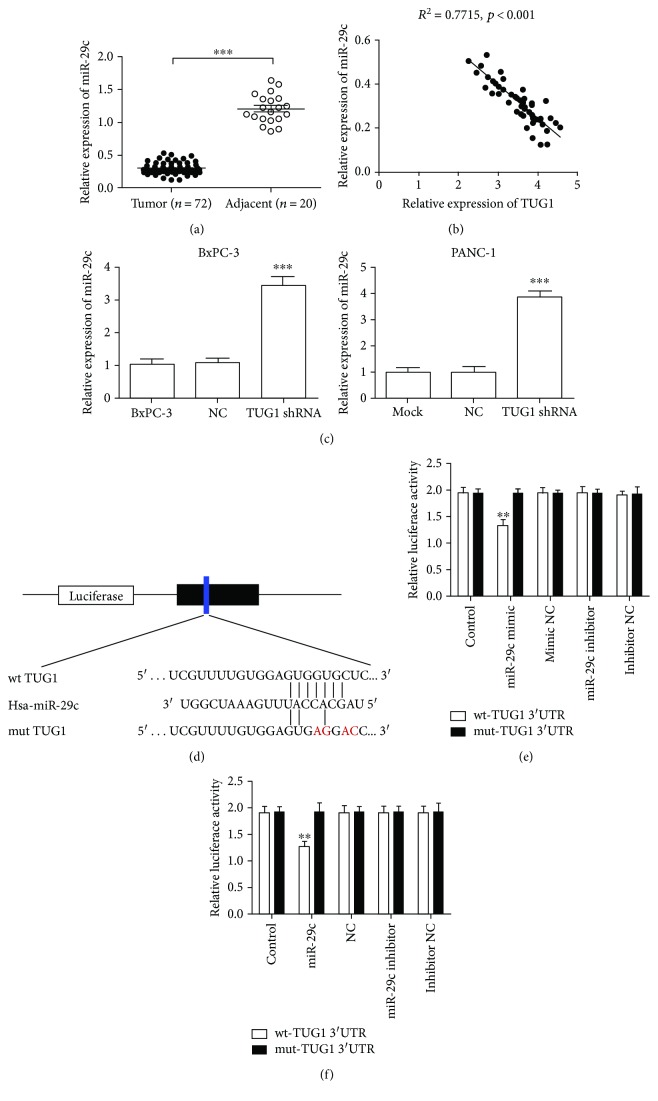
miR-29c was downregulation in PC tissues and bound to TUG1. (a) The expression of miR-29c in human pancreatic cancer tissues was significantly lower than that in peritumoural normal tissues by the qRT-PCR method. (b) Correlation between TUG1 and miR-29c in pancreatic cancer tissues. (c) qRT-PCR was performed to determine the expression of miR-29c in BxPC-3 and PANC-1 cells after Lv-TUG1 shRNA transfection. (d) The predicted binding sequences of miR-29c in TUG1. Mutation was generated in the seed region (red bases) of TUG1. (e, f) The relative luciferase activity was inhibited in BxPC-3 (e) and PANC-1 (f) cells cotransfected with wild-type (wt) TUG1 3′UTR and miR-29c; however, the relative luciferase activity was not inhibited in cells transfected with mutant-type (mut) TUG1 3′UTR. Firefly luciferase activity was normalised to Renilla luciferase. ^∗∗^ *P* < 0.01 vs. NC group.

Bioinformatics analysis was used to identify the potential targeted microRNAs of TUG1 (http://starbase.sysu.edu.cn/). A binding site for miR-29c was found in the TUG1 transcript, and TUG1 was the predicted gene of miR-29c. Initially, we examined the expression of miR-29c at the tissue level. The expression of miR-29c in PC tissues was significantly lower than that in adjacent tissues (Figure 3(a)); this finding was contrary to the expression of TUG1. Moreover, the expression of miR-29c showed no correlation with age (*P* = 0.547), gender (*P* = 0.525), and tumour size (*P* = 0.509). However, it was significantly associated with lymph node metastasis (P = 0.01), pathological differentiation (*P* = 0.042), and clinical stage (*P* = 0.039). We further analysed the correlation between TUG1 and miR-29c in PC tissues. The results showed a negative correlation between the expressions of miR-29c and TUG1 (*R* ^2^ = 0.7715, *P* < 0.001, Figure 3(b)). The expression of miR-29c was dramatically upregulated after TUG1 knockdown in BxPC-3 and PANC-1 cells (Figure 3(c)). These results indicate the target-regulatory relationship between TUG1 and miR-29c. To investigate whether the predicted binding site of miR-29c to 3′UTR of TUG1 is responsible for this regulation, we cloned the 3′UTR of TUG1 downstream to a luciferase reporter gene (wt-TUG1 3′UTR); its mutant version (mut-TUG1 3′UTR) was also constructed by site-directed mutagenesis (Figure 3(d)). The luciferase activity of cells cotransfected with miR-29c mimics and wt-TUG1 3′UTR was significantly reduced compared with that of scramble control cells. Moreover, the miR-29c-mediated repression of luciferase activity was abolished by the mutant putative binding site in BxPC-3 and PANC-1 cells (Figures 3(e) and 3(f)).

### 3.4. Downregulation of miR-29c Abolishes the TUG1 Knockdown-Mediated Inhibition of Tumour Growth In Vitro and In Vivo

**Figure 4:**
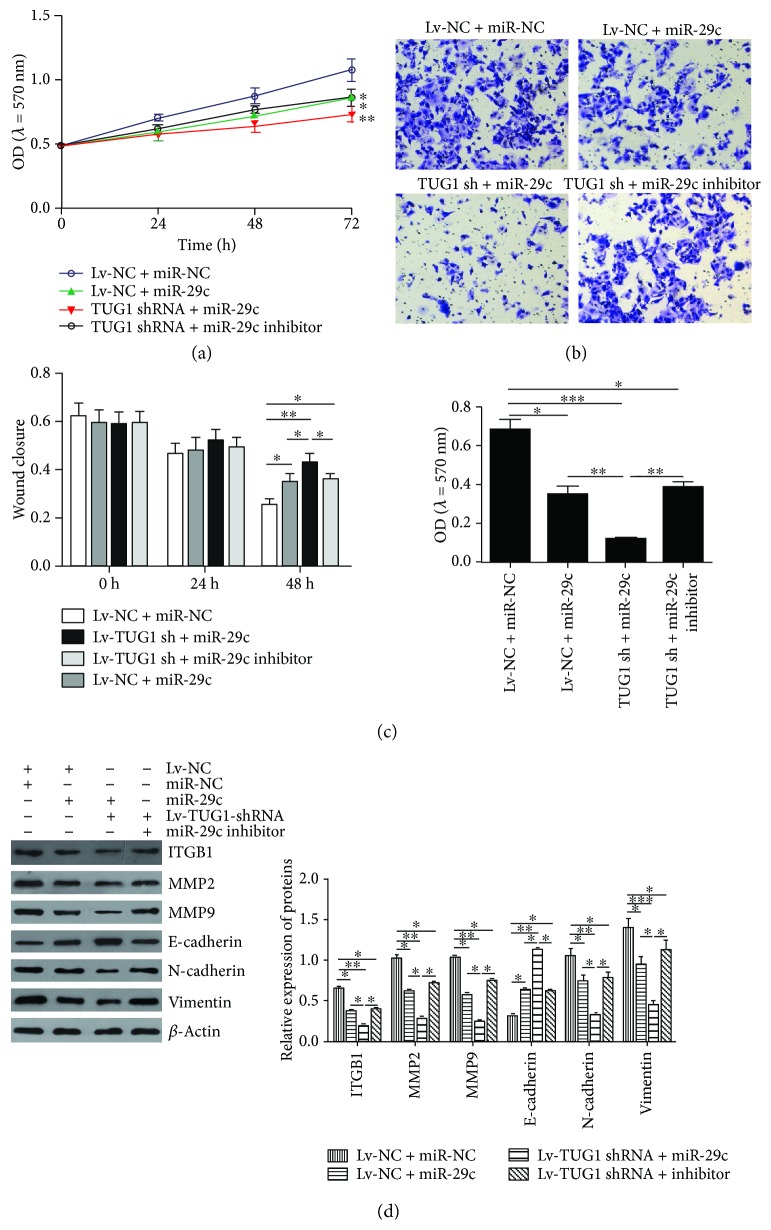
Downregulation of miR-29c abolishes the TUG1 knockdown-mediated inhibition of the proliferative and invasive abilities of BxPC-3 and PANC-1 cells. (a) CCK-8 was used to measure the proliferation of PANC-1 cells after the indicated treatment. (b) Transwell assay was used to measure the invasive ability of PANC-1 cells after the indicated treatment. (c) Wound healing assay was used to measure the migratory ability of PANC-1 cells after the indicated treatment. (d) Expressions of ITGB1, MMP2, MMP9, and EMT marker factors (E-cadherin, N-cadherin, and Vimentin) were measured by Western blot after the indicated treatment (left) and then quantified (right). Data expressed as mean ± SD. ^∗^ *P* < 0.05 and ^∗∗^ *P* < 0.01.

**Figure 5:**
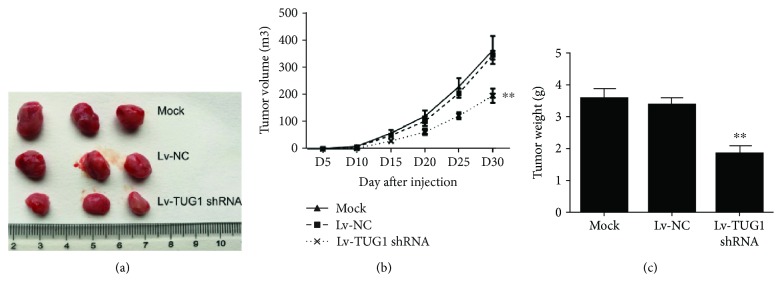
TUG1 knockdown suppresses pancreatic carcinoma growth in vivo. (a) Xenografted tumour obtained from nude mice. (b) Tumour volume was measured every 5 days. (c) Tumour weights. Data are expressed as mean ± SD. ^∗∗^ *P* < 0.01 vs. negative control.

We further investigated the underlying mechanism by which TUG1 inhibits PC cell proliferation and invasion. PANC-1 cells were transfected with shRNA-TUG1 and miR-29c mimic or inhibitor. As shown in Figures 4(a) and 4(c), the restoration of miR-29c expression adequately enhanced the inhibitory effects of TUG1 knockdown on cell proliferation, invasion, and migration, whereas the downregulation of miR-29c reversed the effects of TUG1 knockdown on PANC-1 cells. Furthermore, the downregulation of TUG1 significantly reduced the expressions of ITGB1, MMP2, MMP9, and mesenchymal markers such as N-cadherin and Vimentin, but increased the expression of epithelial marker E-cadherin, which was abolished by the inhibition of miR-29c (Figure 4(d)). We further confirmed the role of TUG1 in vivo. As shown in Figures 5(a)–5(c), tumour growth was significantly suppressed by TUG1 knockdown. These findings demonstrate that the inhibitory effects of TUG1 knockdown on PC progression are achieved via miR-29c.

## 4. Discussion

In this study, we observed that TUG1 is highly expressed in PC tissues compared with that in peritumoural normal tissues. A higher TUG1 level was associated with lymph node metastasis, pathological differentiation, and clinical stage, and the high expression of TUG1 was associated with the poor prognosis of patients with PC. Furthermore, TUG1 knockdown induced significant inhibition of growth, invasion, and migration ability of BxPC-3 and PANC-1 cells.

The upregulation of TUG1 has been demonstrated in several types of cancers, such as gastric cancer and glioma [14, 15]. For example, the TUG1 level in clear cell renal cell carcinoma (ccRCC) tissues was significantly higher than that in adjacent nontumour tissues. A higher TUG1 expression level was associated with the shorter overall survival of patients with ccRCC and was shown to be an independent predictor of poor outcomes [16, 17]. TUG1 knockdown suppressed cell growth, proliferation, and invasion and also induced the apoptosis of oral squamous cell carcinoma by targeting Wnt/*β*-catenin signalling [18]. Elevated TUG1 expression was shown to correlate with larger tumour size, the advanced stage of the International Federation of Gynecology and Obstetrics, poor differentiation, and lymph node metastasis in patients with cervical cancer [19]. Furthermore, the silencing of TUG1 inhibited cell migration and invasion via the inhibition of EMT in cervical cancer cells [19]. Interestingly, Niu et al. [20] found that TUG1 was overexpressed in small-cell lung carcinoma (SCLC) tissues, and its expression levels showed a correlation with clinical stage and shorter survival time of patients with SCLC. Moreover, the downregulation of TUG1 expression impaired cell proliferation and increased the sensitivity of cancer cells to anticancer drugs both in vitro and in vivo [20]. However, Zhang et al. [11] found that TUG1 was generally downregulated in NSCLC tissues and that the lower expression of TUG1 was associated with a higher TNM stage and tumour size, as well as poorer overall survival. As a direct transcriptional target of p53, TUG1 knockdown significantly promotes cell proliferation in vitro and in vivo [11]. Thus, the functions of TUG1 in the context of tumourigenesis are cell- and tissue-specific.

We observed that TUG1 directly targeted miR-29c, a tumour suppressor in several cancers [21–23]. Furthermore, the downregulation of miR-29c abolished the TUG1 knockdown-mediated inhibition of tumour growth in vitro and in vivo; conversely, the upregulation of miR-29c enhanced the effects of TUG1 knockdown on PC cells. Jiang et al. [24] found a significant downregulation of miR-29c in PC tissues due to the relative hyperactivation of Wnt cascade. miR-29c directly suppressed the Wnt upstream regulators. The expression level of miR-29c was shown to be associated with the survival time of patients with PC. Increased miR-29c suppressed cell migration and invasion in vitro and in vivo by targeting MMP2 [25]. Furthermore, Lu et al. [13] demonstrated that miR-29c is frequently downregulated in clinical PC tissues and cell lines. The overexpression of miR-29c significantly inhibited the proliferation, migration, and invasion of PC cells in vitro, suggesting that miR-29c acts as a tumour suppressor in PC cells. They also revealed that ITGB1 was one of the functional target genes of miR-29c, and the effects of ITGB1 knockdown were similar to those of miR-29c overexpression [13]. A previous study showed that altered ITGB1 expression had a significant correlation with lymph node metastasis and depth of invasion in colorectal cancer [26]. The knockdown of ITGB1 inhibited cell adhesion, migration, and proliferation on types I and IV collagen, fibronectin, and laminin in vitro and in vivo on PC [27]. In line with these findings, the knockdown of TUG1 in the present study significantly increased the expression of miR-29c, which in turn induced the downregulation of ITGB1, MMP2, and MMP9. MMPs mediate the basement membrane breach to allow the movement of cells through tissues. The targeting of MMPs has been shown to play a major role in the regulation of cancer cell metastasis [28]. Recent studies have shown a correlation between increased MMP2/MMP9 gene expression in tumour tissues and clinical status, histopathological grading, and metastasis occurrence [29, 30]. The regulatory effect of TUG1 on cancer cell migration and invasion involves the progression of EMT [19]. Our results showed that TUG1 knockdown significantly increased the expression of E-cadherin but suppressed the mesenchymal markers N-cadherin and Vimentin, whereas the miR-29c inhibitor attenuated this effect. These findings indicate that the inhibitory effect of TUG1 knockdown on PC cell invasion is also associated with EMT progression.

## 5. Conclusion

Our study demonstrated the oncogenic role of TUG1 in PC. The downregulation of TUG1 significantly inhibited the growth and migratory activity of PC cells both in vitro and in vivo by targeting miR-29c. Our study provides a novel potential diagnostic biomarker and therapeutic target for PC.

## Figures and Tables

**Table 1:** Clinicopathological association of TUG1 and miR-29c expression in pancreatic cancer patients.

Clinicopathological features	No. of cases	TUG1 expression		*P* value	miR-29c expression		*P* value
		High	Low		High	Low	
*Age (years)*				0.571			0.547
>60	42	29	13		19	23	
≤60	30	21	9		14	16	
*Gender*				0.253			0.525
Female	27	17	10		12	15	
male	45	33	12		21	24	
*Tumour size*				0.159			0.509
>5 cm	25	15	10		11	14	
≤5 cm	47	35	12		22	25	
*Clinical stage*				0.016			0.039
I + II	48	29	19		18	30	
III + IV	24	21	3		15	9	
*Lymph node status*				0.019			0.01
Metastasis	41	33	8		12	29	
No metastasis	31	17	14		21	10	
*Pathological differentiation*				0.032			0.042
Well	39	23	16		22	17	
Moderately-poorly	33	27	6		11	22	

## Data Availability

The data used to support the findings of this study are available from the corresponding author upon request.

## Supplementary Materials

Supplementary Materials Figure S1: enhancing of TUG1 expression promotes the growth, invasion, and migration in SW1990 cells. (A) qRT-PCR was used to determine the expression of lncRNA TUG1 in SW1990 cells after TUG1-pcDNA transfection. (B) CCK-8 was used to measure the proliferation of SW1990 cells after TUG1-pcDNA transfection. (C) Transwell assay was used to measure the invasive ability of SW1990 cells after TUG1-pcDNA transfection. (D) Wound healing assay was used to measure the migratory ability of SW1990 cells after TUG1-pcDNA transfection. Data expressed as mean ± SD. ^∗^ *P* < 0.05, ^∗∗^ *P* < 0.01, and ^∗∗∗^ *P* < 0.001.
