# Corrigendum to “Long Noncoding RNA TUG1/miR-29c Axis Affects Cell Proliferation, Invasion, and Migration in Human Pancreatic Cancer”

**DOI:** 10.1155/2021/5150272

**Published:** 2021-05-19

**Authors:** Yebin Lu, Ling Tang, Zhipeng Zhang, Shengyu Li, Shuai Liang, Liandong Ji, Bo Yang, Yu Liu, Wei Wei

**Affiliations:** ^1^Department of General Surgery, Xiangya Hospital, Central South University, Changsha 410008, China; ^2^Department of Pharmacy, Xiangya Hospital, Central South University, Changsha 410008, China; ^3^Department of Vascular Surgery, Tianjin First Center Hospital, 300192, China; ^4^Department of Pathology, Hunan Provincial People's Hospital, Changsha 410005, China

In the article titled “Long Noncoding RNA TUG1/miR-29c Axis Affects Cell Proliferation, Invasion, and Migration in Human Pancreatic Cancer” [1], BLASTn results [2] identified an error in the forward and reverse sequences of the TUG1 shRNA shown in Section 2.2 of the Materials and Methods Section. The reverse sequence of the TUG1 shRNA reported in the original article was identified as targeting the PMM2 gene sequence; however, the authors have confirmed that it was a mistake, and the correct sequences are as follows:

Correct forward sequence should read as follows:

5′-gatccGCTTGGCTTCTATTCTGAATCCTTTCAAGAGAAGGATTCAGAATAGAAGCCAAGCTTTTTTG-3′.

Correct reverse sequence should read as follows:

5′-CAAAAAAGCTTGGCTTCTATTCTGAATCCTTCTCTTGAAAGGATTCAGAATAGAAGCCAAGCG-3′.
