# Supplementary figures and images for: Long Noncoding RNA TUG1/miR-29c Axis Affects Cell Proliferation, Invasion, and Migration in Human Pancreatic Cancer
Source: Dis Markers. 2018 Nov 22;2018:6857042. doi: 10.1155/2018/6857042 (PMC6282130; doi:10.1155/2018/6857042)

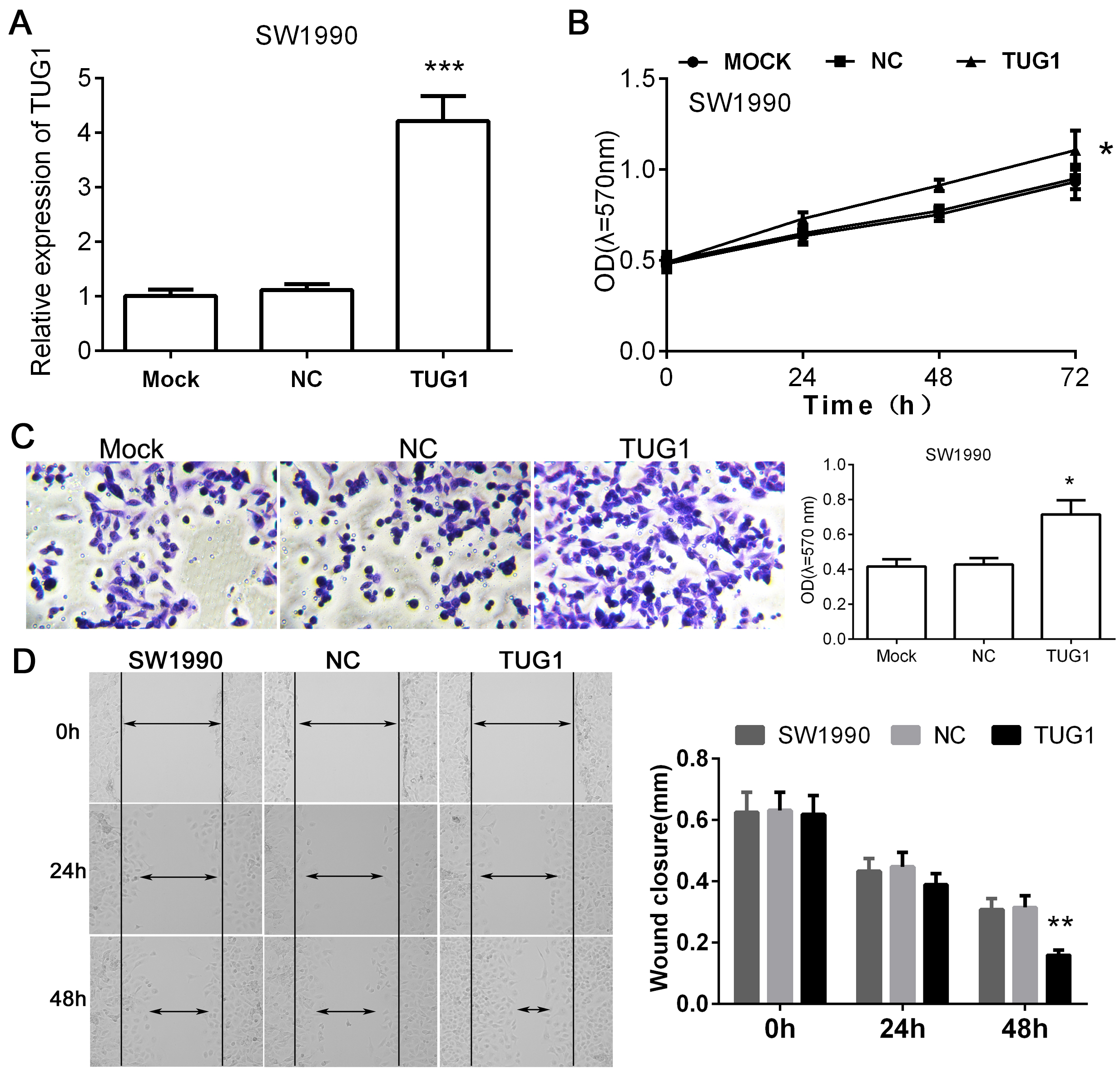

Supplement: Supplementary Materials — Figure S1: enhancing of TUG1 expression promotes the growth, invasion, and migration in SW1990 cells. (A) qRT-PCR was used to determine the expression of lncRNA TUG1 in SW1990 cells after TUG1-pcDNA transfection. (B) CCK-8 was used to measure the proliferation of SW1990 cells after TUG1-pcDNA transfection. (C) Transwell assay was used to measure the invasive ability of SW1990 cells after TUG1-pcDNA transfection. (D) Wound healing assay was used to measure the migratory ability of SW1990 cells after TUG1-pcDNA transfection. Data expressed as mean ± SD. ∗ P < 0.05, ∗∗ P < 0.01, and ∗∗∗ P < 0.001. [file 6857042.f1.jpg]
